# Optimization and validation of a micro–QuEChERS method for phthalates detection in small samples of cetacean blubber^✰^

**DOI:** 10.1016/j.mex.2023.102502

**Published:** 2023-11-29

**Authors:** Annalisa Sambolino, Marta Rodriguez, Jesus De la Fuente, Manuel Arbelo, Antonio Fernández, Manfred Kaufmann, Nereida Cordeiro, Ana Dinis

**Affiliations:** a MARE - Marine and Environmental Sciences Centre, ARNET - Aquatic Research Network, Regional Agency for the Development of Research, Technology and Innovation (ARDITI), Funchal, Portugal; b LB3, Faculty of Exact Science and Engineering, University of Madeira, Funchal, Portugal; c Faculty of Life Sciences, University of Madeira, Funchal, Portugal; d Division of Histology and Animal Pathology, University Institute for Animal Health and Food Security (IUSA), Veterinary School, University of Las Palmas de Gran Canaria, Canary Islands, Spain; e CIIMAR-Interdisciplinary Centre of Marine and Environmental Research, 4450-208 Matosinhos, Portugal

**Keywords:** Bottlenose dolphin, Pilot whale, Phthalic acid esters, Plastic additives, GC–MS, Gas chromatography-mass spectrometry, Ammonium formate “Micro – QuEChERS” method for phthalates detection in small quantities of cetacean blubber

## Abstract

In this study, an innovative method was developed to detect and quantify phthalates in fresh cetacean blubber. An adaptation of the ammonium formate QuEChERS method was used and adapted as a micro-extraction for small quantities of samples. Significantly, this technique utilized minimal quantities of reagents and salts, with the additional implementation of rigorous Quality Assurance/Quality Control protocols to further reduce background contamination. To ensure the reliability of this method, comprehensive validation procedures were conducted, with a specific focus on two widely studied cetacean species: the common bottlenose dolphin (*Tursiops truncatus*) and the short-finned pilot whale (*Globicephala macrorhynchus*). Determination coefficients (R^2^) for matrix-matched calibration were >0.93 with limits of quantifications (LOQ) of the method in the range of 5–10 ng/g. Mean recovery values were between 40 and 100 %. This novel methodology holds particular relevance for environmental research studies, offering the capability to detect emerging contaminants with minimal sample requirements. This aspect is particularly valuable in investigations that involve free-ranging animals and rely on biopsy sampling. It allows for the assessment of contaminant levels in healthy individuals within wild populations, enhancing our understanding of ecological impacts and potential conservation measures.

• A micro-extraction adaptation of the ammonium formate QuEChERS method was developed and applied to a small quantity of fresh cetacean blubber to detect phthalates.
• Small quantities of reagents and salts were used, and additional Quality Assurance/ Quality Control procedures were taken to further minimize background contamination.
• Method validation was carried out for two cosmopolitan and extensively studied cetacean species: the common bottlenose dolphin (*Tursiops truncatus*) and the short-finned pilot whale (*Globicephala macrorhynchus*).

## Graphical abstract

**Figure:**
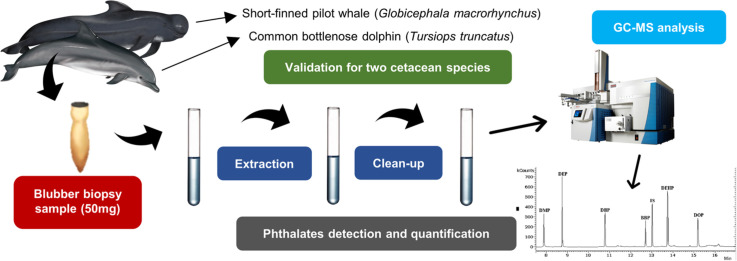

Specifications table

**Table:** 

Subject area:	Environmental Science
More specific subject area:	Environmental Analytical Chemistry
Name of your method:	Ammonium formate “Micro – QuEChERS” method for phthalates detection in small quantities of cetacean blubber
Name and reference of the original method:	Sambolino, A., Ortega-Zamora, C., González-Sálamo, J., Dinis, A., Cordeiro, N., Canning-Clode, J. and Hernández-Borges, J., 2022. Determination of phthalic acid esters and di (2-ethylhexyl) adipate in fish and squid using the ammonium formate version of the QuEChERS method combined with gas chromatography mass spectrometry. Food Chemistry, 380, p.132174. https://doi.org/10.1016/j.foodchem.2022.132174
Resource availability:	Not applicable

## Method details

A modified version of the QuEChERS method described in Sambolino et al. [1] was applied to a small quantity of cetacean blubber for phthalates extraction and purification (named micro- QuEChERS). The present method, in order to be applied in 100x smaller quantity of tissue, sees a considerably reduced amount of extraction reagents, while maintaining the same amount of purification salts, given the higher fat concentrations of the target tissue. An extra step was introduced to concentrate the final extract, given the limited amount of analytes extractable from such small samples. For this reason, additional cleaning procedures were implemented to control background contamination, minimizing the risk of co-extraction of phthalates in reagents and glassware. The target analytes in this modified approach are seven phthalates which are the most commonly encountered in the marine environment [2], [3], [4].

Blubber tissue was collected from stranded cetaceans (common bottlenose dolphin - *Tursiops truncatus* and short-finned pilot whale - *Globicephala macrorhynchus*) and kept frozen at −20° C until the day of analysis. On the analysis day, a small portion of sample (50 mg, wet weight [w.w.]) was cut, added to a 15 mL glass tube, and mashed, still frozen, with a glass rod for at least 1 min. One ml of Acetonitrile (ACN) was then added to the glass tube with the freeze-homogenized sample, vortexed for 1 min, then 0.5 g of ammonium formate was added, in order to induce phase separation [5]; the mixture was vortexed again for 1 min and then centrifuged for 5 min at 2500 rpm. For the purification step, all the supernatant was transferred to a glass tube containing 150 mg of MgSO_4_, 50 mg of Primary Secondary Amine (PSA), and 50 mg of C18. The ratio of ammonium formate to ACN and MgSO_4_ in PSA and C18 followed established methodologies [1,5], although quantities of reagents were considerably reduced in comparison to the original method [1]. The mixture was again vortexed for 1 min and centrifuged for 5 min at 2500 rpm. The resulting supernatant (200 µl) was transferred to a GC vial, evaporated overnight, and then reconstituted in 50 µl of Cyclohexane (CH).

To minimize contamination, all the glassware was carefully cleaned, immersed in an acid bath for 24 h, and then muffled at 550° C overnight [1,6]. No plastic material besides pipette tips (phthalate-free) was used during the procedure. To minimize background contamination from organic reagents, ACN and CH were double distilled before use [7] and salts were washed three times with methanol. Two procedural blanks were analyzed with each batch of samples, and an average of the two blank values (RSD < 10 %) was subtracted from the final results. High-purity solvents and reagents were used. Methanol, ACN and CH of LC-MS grade, ammonium formate (purity ≥ 97.0 %) and MgSO_4_ (purity ≥ 98.0 %) were from VWR International Eurolab (Barcelona, Spain). PSA and C18 were from Agilent Technologies (Santa Clara, CA, USA).

Seven phthalates (Phthalic Acid Ester - PAE) were the target analytes (chemical structures and proprieties in Table 1). High purity standards (>98 %) of each PAE and two isotopically labeled PAEs (DEP-D4 and DBP-D4, used as internal standards) were acquired from Sigma-Aldrich (Madrid, Spain) and Dr. Ehrenstorfer (Augsburg, Germany). The determination and quantification of the analytes was carried out with an Agilent 6890 GC (Agilent Technologies, California, USA), equipped with an Ultra Inert HP-5 ms capillary column (30 m × 0.25 mm inner diameter, 0.25 µm film thickness), coupled with an Agilent 5973 Network MS (Agilent Technologies, California, USA), in the selected ion monitoring (SIM) mode. The oven temperature program started at 60 °C (1 min), then increased to 190 °C at 45 °C/min, and finally increased to 310 °C at 35 °C/min and held for 3 min. Injection was in splitless mode (purge time 0.75 min; purge flow 40 ml/min) at 280 °C, with an injection volume of 2 µl. The detection parameters follow those described in Sambolino et al. [1]; retention times, ion qualifiers and quantifiers of selected PAEs for this study are provided in Table 2.

**Table 1:** Chemical structure and properties of the studied phthalates.

Analyte	Structure	Molecular formula	MM (g/mol)	Solubility in water (g/L, 25 °C)	Vapor pressure (mmHg, 25 °C)	Log K_OW_	Melting point (°C)	Boiling point (°C)
DMP		C_10_H_10_O_4_	194.2	4.3	3.08·10^−3^	1.60	5.5	284
DEP		C_12_H_14_O_4_	222.2	1.08	2.1·10^−3^	2.47	−3	295
DIBP		C_16_H_22_O_4_	278.3	0.0062^a^	4.76·10^−5^	4.11	−37	320
DBP		C_16_H_22_O_4_	278.2	0.0112	2.01·10^−5^	4.72	−35	340
BBP		C_19_H_20_O_4_	312.1	0.00269	8.25·10^−6^	4.73	−35	370
DEHP		C_24_H_38_O_4_	390.3	0.00027	1.42·10^−7^	7.60	−55	384
DNOP		C_24_H_38_O_4_	390.6	0.000022	1.0·10^−7^	8.20	−25	385

a 24 °C. Data taken from SciFinder® and PubChem databases. MM: Molecular mass.

**Table 2:** Retention times and m/z values of quantifier and qualifier ions in GC–MS analyses of the target analytes and internal standards (in bold).

Analyte	Retention time (min)	Quantifier (m/z)	Qualifier 1 (m/z)	Qualifier 2 (m/z)
DMP	4.678	163	77	194
**DEP-d_4_**	5.090	153	181	80
DEP	5.096	149	177	76
DIBP	5.872	149	223	104
**DBP-d_4_**	6.122	153	209	227
DBP	6.126	149	205	223
BBP	7.152	149	91	206
DEHP	7.559	149	167	279
DNOP	8.028	149	167	279

- Ionization energy of 70 eV in all cases.

## Method validation

The modified QuEChERS method (micro-QuEChERS) was validated through several steps. First, an instrument calibration using the internal standard approach was done in order to quantify the blank values and calculate the matrix effect (Table 3). Recoveries studies at two spiking levels (25 and 150 ng/g w.w.) and matrix-matched calibrations with the internal standard method were then carried out for both matrices (blubber samples from the two species) (Tables 4 and 5). The equations coefficient obtained were used to quantify the compounds in the analyzed samples. The studied linear range, the matrix effect (ME) and the limits of quantifications (LOQ) of the method, considered as the lowest calibration level with signal to noise ratio > 10, are reported in Table 5 for the two different matrices analyzed.

**Table 3:** Internal instrumental calibration data of the target analytes.

Analyte	Studied linear range (µg/L)	Regression equation (*n* = 8)		s_y/x_	R^2^
		*b* ± s_b_·t_(0.05;7)_	*a* ± s_a_·t_(0.05;7)_		
DMP	5–300	(5.37 ± 0.49)·10^−3^	(0.54 ± 6.7)·10^−2^	4.67·10^−2^	0.9938
DEP	5–300	(7.68 ± 0.72)·10^−3^	(7.64 ± 9.84)·10^−2^	6.86·10^−2^	0.9934
DIBP	5–300	(10.19 ± 1.24)·10^−3^	(4.06 ± 17.57)·10^−2^	10.92·10^−2^	0.9924
DBP	5–300	(11.68 ± 1.05)·10^−3^	(1.31 ± 14.43)·10^−2^	10.06·10^−2^	0.9939
BBP	5–300	(4.19 ± 0.42)·10^−3^	(5.88 ± 6.57)·10^−2^	3.11·10^−2^	0.9970
DEHP	5–300	(6.44 ± 0.73)·10^−3^	(3.22 ± 10.65)·10^−2^	5.39·10^−2^	0.9962
DNOP	5–300	(9.42 ± 1.04)·10^−3^	(1.52 ± 16.36)·10^−2^	7.74·10^−2^	0.9964

b: slope; S_b_: standard error of the slope; t_(0.05;7)_: t-multiplier for 95 % confidence interval calculation; a: intercept; S_a_: standard error of the intercept; s_y/x_: standard error of the estimate; R^2^: determination coefficient.

**Table 4:** Relative recovery (%) and RSD values (in brackets) of the target analytes from recovery studies with two spiking levels on blubber samples.

Matrix (Species)	Analyte	Level 1	Level 2	Mean
Short-finned pilot whale (*Globicephala macrorhynchus*)	DMP	88 (**37**)	108 (**43**)	100 (**38**)
	DEP	**61** (**30**)	**121** (**41**)	97 (**51**)
	DIBP	72 (5)	103 (4)	88 (20)
	DBP	**59** (6)	74 (3)	**67** (13)
	BBP	**64** (5)	**41** (9)	**53** (**24**)
	DEHP	70 (9)	**37** (10)	**53** (**35**)
	DNOP	**162** (13)	**23** (14)	93 (**83**)
Bottlenose dolphin (*Tursiops truncatus*)	DMP	**45 (21)**	**36** (20)	**40 (22)**
	DEP	82 (**22**)	92 (6)	87 (15)
	DIBP	83 (**28)**	88 (8)	85 (18)
	DBP	92 (20)	107 (11)	100 (16)
	BBP	114 (**31**)	78 (7)	92 (**28**)
	DEHP	110 (20)	83 (3)	97 (**21**)
	DNOP	75 (4)	**57** (11)	**65** (17)

Level 1: 25 ng/g of w.w; level 2: 150 ng/g of w.w. Data outside the 70–120 % range for recovery values and 0–20 % for RSD values are in bold.

**Table 5:** Matrix-matched calibration data of the selected PAEs, with limits of quantification (LOQ) and matrix effect (ME) percentage in cetacean blubber samples.

Matrix (species)	Analyte	Studied linear range (ng/g)	Regression equation (*n* = 8)		s_y/x_	R^2^	LOQ (ng/g)*	ME (%)**
			*b* ± s_b_·t_(0.05;7)_	*a* ± s_a_·t_(0.05;7)_				
Short-finned pilot whale (*Globicephala macrorhynchus*)	DMP	5–300	(4.39 ± 1.58)·10^−3^	(0.81 ± 11.34)·10^−2^	0.59·10^−1^	0.9631	10	−18
	DEP	5–300	(8.27 ± 2.14)·10^−3^	(0.61 ± 31.68)·10^−2^	1.72·10^−1^	0.9664	10	8
	DIBP	5–300	(10.9 ± 1.29)·10^−3^	(0.37 ± 17.56)·10^−2^	1.29·10^−1^	0.9894	5	7
	DBP	5–300	(7.84 ± 3.94)·10^−3^	(0.55 ± 57.51)·10^−2^	2.91·10^−1^	0.9302	5	−33
	BBP	5–300	(3.07 ± 0.24)·10^−3^	(0.32 ± 1.94)·10^−2^	0.14·10^−1^	0.9953	10	−27
	DEHP	5–300	(5.8 ± 3.98)·10^−3^	(0.73 ± 64.76)·10^−2^	2.03·10^−1^	0.9515	5	−10
	DNOP	5–300	(9.3 ± 0.4)·10^−3^	(0.71 ± 4.97)·10^−2^	0.39·10^−1^	0.9986	10	−1
Bottlenose dolphin (*Tursiops truncatus*)	DMP	5–300	(7.62 ± 1.11)·10^−3^	(0.14 ± 13.86)·10^−2^	1.09·10^−1^	0.9842	5	42
	DEP	5–300	(8.27 ± 5.02)·10^−3^	(0.35 ± 86.33)·10^−2^	2.52·10^−1^	0.9617	5	8
	DIBP	5–300	(12.02 ± 2.04)·10^−3^	(0.3 ± 31.41)·10^−2^	1.57·10^−1^	0.9915	5	18
	DBP	5–300	(11.38 ± 9.4)·10^−3^	(0.28 ± 145.43)·10^−2^	5.25·10^−1^	0.9314	5	−3
	BBP	5–300	(5.73 ± 0.52)·10^−3^	(0.19 ± 6.7)·10^−2^	0.55·10^−1^	0.9917	10	37
	DEHP	5–300	(6.9 ± 1.34)·10^−3^	(0.42 ± 20.7)·10^−2^	1.01·10^−1^	0.9889	5	7
	DNOP	5–300	(13.96 ± 1.37)·10^−3^	(0.18 ± 17.57)·10^−2^	1.46·10^−1^	0.9904	10	48

b: slope; S_b_: standard deviation of the slope; t_(0.05;7)_: t-multiplier for 95 % confidence interval calculation; a: intercept; S_a_: standard deviation of the intercept; s_y/x_: standard deviation of the estimate; R^2^: determination coefficient. *Calculated as the lowest calibration level with S/*N*>10; **Calculated following the equation used by Kwon et al. (Kwon, Lehotay, & Geis-Asteggiante, 2012). [https://doi.org/10.1016/j.chroma.2012.10.059].

The method yielded recoveries with relatively high variation. For *T. truncatus* samples, mean relative recoveries were between 85–100% for five of the seven PAEs; DMP and DNOP held lower recoveries (40 % and 75 %, respectively). For *G. macrorhynchus*, mean recoveries ranged between 53 % and 100% (Table 4). Matrix-matched calibrations with the internal standard method were calculated for each matrix, obtaining linear regression with fitting R^2^ > 0.93. Limits of quantifications (LOQ) of the method ranged between 5 and 10 ng/g w.w. (Table 5). The matrix effect varied depending on the matrix and the analyte, staying within the ± 20 % limits, except for DBP and BBP in *G. macrorhynchus* (−33 % and −27 %, respectively) and DMP, BBP, and DNOP in *T. truncatus* (+42 %, +37 % and +48 % respectively).

Real samples analysis was conducted on portions of biopsies from the two species (*G. macrorhynchus, n* = 15*; T. truncatus, n* = 9*),* longitudinally cut to encompass all the blubber layers. Results on the PAEs concentrations are reported in Table 6 and are further discussed in the related research article.

**Table 6:** Results of the phthalates (PAEs) analysis of cetacean blubber samples from biopsies of short-finned pilot whales (*Globicephala macrorhynchus, n* = 15) and common bottlenose dolphins (*Tursiops truncatus, n* = 9).

**Matrix (species)**	Sample		Analyte (ng/g) wet weight						
			DMP	DEP	DIBP	DBP	BBP	DEHP	DNOP
Short-finned pilot whale (*Globicephala macrorhynchus*)	Gma46		n.d.	**66.53 ± 27.47**	n.d.	n.d.	<LOQ	n.d.	n.d.
	Gma47		18.56 ± 1.53	30.18 ± 3.55	**7.38 ± 1.88**	281.95 ± 9.45	<LOQ	93.63 ± 15.96	<LOQ
	Gma50		<LOQ	37.82 ± 0.58	n.d.	210.27 ± 8.15	<LOQ	74.25 ± 6.67	n.d.
	Gma51		14.94 ± 2.1	**13.48 ± 4.24**	n.d.	n.d.	n.d.	n.d.	n.d.
	Gma52		n.d.	<LOQ	n.d.	88.98 ± 8.58	<LOQ	**28.76 ± 13.98**	n.d.
	Gma54		<LOQ	27.89 ± 3.74	n.d.	66.28 ± 7.64	<LOQ	n.d.	**13.37 ± 2.83**
	Gma56		<LOQ	93.24 ± 4.6	n.d.	116.76 ± 8.01	<LOQ	29.5 ± 3.91	n.d.
	Gma57		n.d.	404.3 ± 7.23	n.d.	172.48 ± 5.91	<LOQ	n.d.	n.d.
	Gma58		17.73 ± 0.58	63.12 ± 2.14	n.d.	39.76 ± 2.56	<LOQ	n.d.	n.d.
	Gma59		<LOQ	60.25 ± 4.31	n.d.	148.1 ± 5.45	n.d.	n.d.	n.d.
	Gma60		14.47 ± 0.52	69.56 ± 7.04	n.d.	121.58 ± 2.39	10.41 ± 0.87	n.d.	n.d.
	Gma61		12.82 ± 0.84	33.11 ± 6.17	19.41 ± 0.44	284.11 ± 1.77	<LOQ	**14.46 ± 10.59**	n.d.
	Gma62		25.98 ± 0.86	18.6 ± 1.63	n.d.	212.4 ± 10.68	<LOQ	**34.95 ± 9.42**	n.d.
	Gma63		<LOQ	<LOQ	n.d.	n.d.	n.d.	n.d.	n.d.
	Gma64		n.d.	<LOQ	n.d.	258.38 ± 3.8	<LOQ	n.d.	n.d.
Common bottlenose dolphin (*Tursiops truncatus*)	Tt02		n.d.	n.d.	n.d.	n.d.	15.46 ± 2.38	295.56 ± 8.28	n.d.
	Tt03		n.d.	38.94 ± 3.08	n.d.	n.d.	<LOQ	127.3 ± 23.71	n.d.
	Tt17		n.d.	n.d.	n.d.	40.1 ± 3.13	**11.32 ± 3.12**	254.99 ± 19.13	n.d.
	Tt47		n.d.	n.d.	n.d.	n.d.	<LOQ	163.49 ± 9.99	n.d.
	Tt48		n.d.	**9.99 ± 4.7**	**28.01 ± 13.18**	134.21 ± 11.89	21.85 ± 2.57	517.77 ± 5.78	n.d.
	Tt49		n.d.	n.d.	57.82 ± 3.72	174.89 ± 1.51	57.11 ± 3.02	4697.34 ± 113.45	n.d.
	Tt50		n.d.	n.d.	n.d.	**47.9 ± 17.88**	14.99 ± 1.93	318.91 ± 3.72	n.d.
	Tt51		n.d.	n.d.	n.d.	n.d.	<LOQ	180.79 ± 14.25	n.d.
	Tt 52		n.d.	27.19 ± 8.48	**92.7 ± 22.21**	717.59 ± 36.51	19.85 ± 1.82	446.97 ± 19.82	n.d.

< LOQ: below the limits of quantification, ½ of the LOQ value was considered for statistical analysis; n.d.: analyte not detected (below limit of detection); values in bold have RSD > 20 %.

## Additional information

The present manuscript proposes a new rapid, cost-effective methodology to analyze phthalates in small blubber samples from two odontocete species. Bottlenose dolphins and pilot whales, extensively studied among delphinid species due to their widespread abundance and distribution in temperate and tropical waters [8,9], face significant anthropogenic impacts, sharing habitats and resources with human activities [1,10]. Conservation efforts necessitate monitoring their contaminant status. The use of biopsy sampling in free-ranging individuals offers a superior assessment of wild population health compared to stranded animals [11,12]. However, these samples are extremely limited in quantity, demanding innovative methodologies for their effective use. To the best of our knowledge, only one other method [13] has been developed for working with such samples. Our refined approach, aligning with QuEChERS extraction principles, proves notably more efficient and faster, utilizing only half the sample quantity (50 mg vs. 100 mg).

Previous studies [14,15] indicate that pollutant concentrations may vary based on blubber layer stratification and sample body location. Therefore, analyzed biopsy samples should encompass all layers, from beneath the skin to above the muscle, with due consideration to the sample's body location.

The proposed method is anticipated to produce comparable results for other odontocete species, however, blubber tissue in different cetacean species exhibits variability in thickness and triglycerides/wax esters composition [16]. Thus, when applying the method to new species, validation is strongly recommended.

Analyzing blubber is challenging due to its high lipid content, posing interference with chromatographic analysis. While employing ACN facilitates lipid removal from fat tissues, an additional cleanup step is essential [17]. Dispersive solid-phase extraction (d-SPE) with a mixture of MgSO4, PSA and C18 was found effective for coextractives removal from acetonitrile extract [18]. However, some coeluates extracted during the procedure may interfere with the final analysis, causing signal suppression [19], which was observed in the matrix effect (Table 5). Recovery rates for most analytes ranged from 80 to 100 %. However, analytes like DMP exhibited a significant loss, potentially attributed to the drying step, resulting in a lower rate of 40 %.

## Declaration of generative AI and AI-assisted technologies in the writing process

During the preparation of this work the authors used ChatGPT in order to improve text fluency and readability. After using this tool, the authors reviewed and edited the content as needed and take full responsibility for the content of the publication.

## CRediT authorship contribution statement

**Annalisa Sambolino:** Conceptualization, Methodology, Validation, Formal analysis, Investigation, Data curation, Writing – original draft, Writing – review & editing, Visualization. **Marta Rodriguez:** Validation, Formal analysis, Investigation, Data curation, Writing – review & editing. **Jesus De la Fuente:** Conceptualization, Resources, Project administration, Funding acquisition, Writing – review & editing. **Manuel Arbelo:** Resources, Project administration, Funding acquisition, Writing – review & editing. **Antonio Fernández:** Project administration, Funding acquisition. **Manfred Kaufmann:** Resources, Supervision, Project administration, Funding acquisition, Writing – review & editing. **Nereida Cordeiro:** Resources, Conceptualization, Supervision, Project administration, Funding acquisition, Writing – review & editing. **Ana Dinis:** Resources, Conceptualization, Supervision, Project administration, Funding acquisition, Writing – review & editing.

## Declaration of Competing Interest

The authors declare that they have no known competing financial interests or personal relationships that could have appeared to influence the work reported in this paper.

## Data Availability

Data will be made available on request.

## References

[1] Sambolino A., Ortega-Zamora C., González-Sálamo J., Dinis A., Cordeiro N., Canning-Clode J., Hernández-Borges J. (2022). Determination of phthalic acid esters and di (2-ethylhexyl) adipate in fish and squid using the ammonium formate version of the QuEChERS method combined with gas chromatography mass spectrometry. Food Chem.

[2] Net S., Sempéré R., Delmont A., Paluselli A., Ouddane B. (2015). Occurrence, fate, behavior and ecotoxicological state of phthalates in different environmental matrices. Environ. Sci. Technol..

[3] Paluselli A., Aminot Y., Galgani F., Net S., Sempéré R. (2018). Occurrence of phthalate acid esters (PAEs) in the northwestern Mediterranean Sea and the Rhone River. Prog. Oceanogr..

[4] Sambolino A., Iniguez E., Herrera I., Kaufmann M., Dinis A., Cordeiro N. (2023). Microplastic ingestion and plastic additive detection in pelagic squid and fish: implications for bioindicators and plastic tracers in open oceanic food webs. Sci. Total Environ..

[5] González-Curbelo M.Á., Lehotay S.J., Hernández-Borges J., Rodríguez-Delgado M.Á. (2014). Use of ammonium formate in QuEChERS for high-throughput analysis of pesticides in food by fast, low-pressure gas chromatography and liquid chromatography tandem mass spectrometry. J. Chromatogr. A.

[6] Paluselli A., Kim S.-K. (2020). Horizontal and vertical distribution of phthalates acid ester (PAEs) in seawater and sediment of East China Sea and Korean South Sea: traces of plastic debris?. Mar. Pollut. Bull..

[7] Notardonato I., Protano C., Vitali M., Avino P. (2019). Phthalates and bisphenol-A determination and release from different beverage plastic containers by dispersive liquid-liquid microextraction and GC-IT/MS analysis. Food Anal. Methods.

[8] Wells R.S., Scott M.D. (2018).

[9] Van Cise A.M., Baird R.W., Baker C.S., Cerchio S., Claridge D., Fielding R., Hancock-Hanser B., Marrero J., Martien K.K., Mignucci-Giannoni A.A. (2019). Oceanographic barriers, divergence, and admixture: phylogeography and taxonomy of two putative subspecies of short-finned pilot whale. Mol. Ecol..

[10] Arranz P., Glarou M., Sprogis K.R. (2021). Decreased resting and nursing in short-finned pilot whales when exposed to louder petrol engine noise of a hybrid whale-watch vessel. Sci. Rep..

[11] Hobbs K.E., Muir D.C., Michaud R., Béland P., Letcher R.J., Norstrom R.J. (2003). PCBs and organochlorine pesticides in blubber biopsies from free-ranging St. Lawrence River Estuary beluga whales (Delphinapterus leucas), 1994–1998. Environ. Pollut..

[12] Fossi M.C., Baini M., Simmonds M.P. (2020). Cetaceans as ocean health indicators of marine litter impact at global scale. Front. Environ. Sci..

[13] Baini M., Martellini T., Cincinelli A., Campani T., Minutoli R., Panti C., Finoia M.G., Fossi M.C. (2017). First detection of seven phthalate esters (PAEs) as plastic tracers in superficial neustonic/planktonic samples and cetacean blubber. Anal. Methods.

[14] Ellisor D., McLellan W., Koopman H., Schwacke L., McFee W., Kucklick J. (2013). The distribution and stratification of persistent organic pollutants and fatty acids in bottlenose dolphin (Tursiops truncatus) blubber. Sci. Total Environ..

[15] Aguilar A., Borrell A. (1991). Heterogeneous distribution of organochlorine contaminants in the blubber of baleen whales: implications for sampling procedures. Mar. Environ. Res..

[16] Koopman H.N. (2007). Phylogenetic, ecological, and ontogenetic factors influencing the biochemical structure of the blubber of odontocetes. Mar. Biol..

[17] Yuan B., Zhao D., Lyu W., Yin Z., Kshatriya D., Simon J.E., Bello N.T., Wu Q. (2021). Development and validation of a micro-QuEChERS method with high-throughput enhanced matrix removal followed with UHPLC-QqQ-MS/MS for analysis of raspberry ketone-related phenolic compounds in adipose tissues. Talanta.

[18] Koesukwiwat U., Lehotay S.J., Mastovska K., Dorweiler K.J., Leepipatpiboon N. (2010). Extension of the QuEChERS method for pesticide residues in cereals to flaxseeds, peanuts, and doughs. J. Agric. Food Chem..

[19] Yu S., Xu X. (2012). Study of matrix-induced effects in multi-residue determination of pesticides by online gel permeation chromatography-gas chromatography/mass spectrometry. Rapid Commun. Mass Spectrom..

